# Assessing Diversity Scaling in Lung Cancer Microbiome Across Individuals and Tissue Types

**DOI:** 10.1002/mbo3.70036

**Published:** 2025-08-17

**Authors:** Jiandong Mei, Yuting Qiao, Zhanshan (Sam) Ma

**Affiliations:** ^1^ Department of Thoracic Surgery, West China Hospital Sichuan University Chengdu China; ^2^ Computational Biology and Medical Ecology Lab, Kunming Institute of Zoology Chinese Academy of Sciences Kunming Yunnan China; ^3^ Kunming College of Life Science University of the Chinese Academy of Sciences Kunming Yunnan China; ^4^ Faculty of Arts and Sciences Harvard University Cambridge Massachusetts USA

**Keywords:** diversity–area relationship (DAR), diversity scaling, human lung cancer microbiome, lung adenocarcinoma (LUAD), lung cancer, lung squamous cell carcinoma (LUSC)

## Abstract

The intra‐tumor microbiome can impact the tumor's behavior by influencing its growth, inflammatory reactions, evasion of the immune system, genomic instability, and drug resistance. Altering this microbiota to improve the response to cancer treatment could offer fresh perspectives on cancer therapy. The very first step in intervening in the microbiome is to gain a deep understanding of how microbial diversity varies spatially and temporally between tissues or among individuals. Such changes can be investigated with the so‐termed diversity–area relationship (DAR) modeling (Ma. 2018. *Ecology and Evolution*, 8(20), 10023–10038). This included examining the DAR profiles, Pairwise Diversity Overlap (PDO) profiles, Maximum Accumulated Diversity (MAD) profiles, and the ratio of local to global accumulated diversity (LGD) profiles. This study applies the DAR approach to reanalyze five lung tissue microbiome datasets to shed light on how the microbial diversity changes across tissue types and across individual patients. We characterized the diversity scaling of human lung cancer microbiota from aspects such as microbial community diversity, variation rates of diversity, similarity, and microbial community proportions. The generated results indicate that there are no statistically significant differences in the DAR scaling parameters across different tissue types, suggesting that the diversity scaling of microbial communities in normal and tumor tissues across individuals seems to be invariant. The invariance is simply a reflection of the resilience of lung tissue microbiome against disturbance such as lung cancer, and thus further studies at the level of microbial species to better understand their relationship with cancer are critical.

## Graphical abstract

Diversity scaling of human lung cancer microbiome was characterized from aspects community diversity, variation rates of diversity, similarity, and community proportions by a diversity–area relationship (DAR) model. DAR parameters of the lung cancer microbiome showed no significant differences across tissue types, indicating invariant diversity scaling in different tissues across individuals.

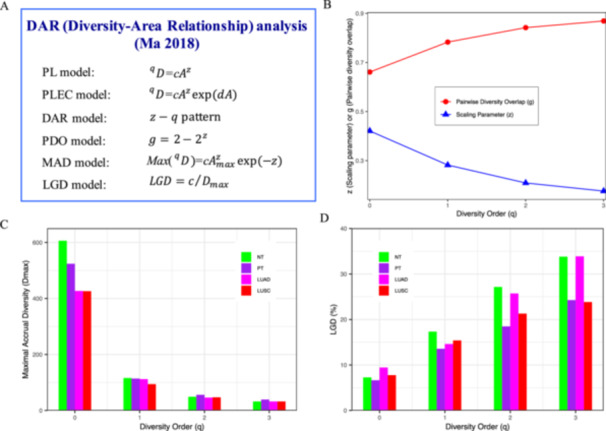

## 1 Introduction

Cancer is a major global health challenge influenced by biotic risk factors—such as viral infections (e.g., human papillomavirus), bacterial pathogens (e.g., *Helicobacter pylori*), microbiome dysbiosis, and chronic inflammation—and abiotic risk factors—including tobacco smoke, ultraviolet radiation, occupational chemicals (e.g., asbestos), and dietary or environmental exposures (e.g., high‐fat diets, air pollution) (Wu et al. 2016; Khan et al. 2024, 2025; Bano et al. 2025). An expanding body of evidence further implicates bacteria in the initiation and progression of diverse cancers: proteins from *Chlamydia pneumoniae* may promote lung carcinogenesis via subcellular targeting (Bano et al. 2025), nuclear‐targeting proteins of *Helicobacter pylori* have been linked to gallbladder cancer development (Wang et al. 2021), Toll‐like receptor signaling modulates prostate tumor growth and offers promising druggable targets (Khan et al. 2025), calcium supplementation intersects with colorectal cancer risk—potentially through effects on the gut microbiota (Khan et al. 2020, 2024)—and predicted interactions of *Mycoplasma hominis* proteins with host organelles further implicate bacterial factors in prostate cancer etiology (Khan et al. 2025).

Lung cancer, the leading cause of cancer‐related deaths worldwide, arises from the malignant transformation of epithelial cells in lung tissue, progressively forming a primary tumor that invades surrounding tissues and metastasizes (Rosell and Karachaliou 2016). The most prevalent form, non‐small cell lung cancer (NSCLC), comprises 85%–88% of cases, while small cell lung cancer (SCLC), known for its rapid growth and metastasis, accounts for 12%–15% (Farver and Zander 2009; Girard et al. 2000). NSCLC is further categorized into subtypes based on histological and treatment characteristics, including adenocarcinoma, squamous cell carcinoma, and large cell carcinoma (Herbst et al. 2008). The etiology of lung cancer is multifactorial, involving complex interactions between genetic predisposition and environmental exposures such as smoking, ionizing radiation, and air pollution (Toyooka et al. 2003; Bade and Dela Cruz 2020; Lemjabbar‐Alaoui et al. 2015).

Microorganisms are increasingly recognized as critical environmental factors in maintaining microecological balance and modulating the host's immune response to therapies (Dickson et al. 2017, 2018). In particular, the lung microbiome directly and indirectly influences host immune activity, shaping susceptibility to various diseases and treatment outcomes (Hanahan and Weinberg 2011; Jin et al. 2019). Tumor development along mucosal surfaces often coincides with the disruption of the immune barrier. Failure to repair such damage leads to chronic inflammation, which can escalate into cancer (Jin et al. 2019; Barrett et al. 2020). Numerous studies have identified microbial populations within tumors and adjacent tissues, suggesting that these microbes, by utilizing tumor‐derived carbon sources, can coexist with and potentially influence the tumor immune microenvironment over prolonged periods (Nejman et al. 2020; Pushalkar et al. 2018; Le Noci et al. 2018).

Disruption of the lung microbiome, known as dysbiosis, has been linked to the activation of carcinogenic pathways via specific microbial mechanisms (Liu et al. 2020). The lung's microbial community, consisting of bacteria, fungi, and viruses, enters through mucosal secretions from the nasopharynx and oropharynx, as well as environmental air exchange. In immunocompetent individuals, the lung microbiome is dominated by several genera, including *Prevotella*, *Streptococcus*, and *Veillonella*, along with other common respiratory‐associated bacteria such as *Neisseria* and *Haemophilus*. These microorganisms, together with fungal species such as *Candida*, *Penicillium,* and *Aspergillus*, are typically present without causing infection in individuals with a healthy immune system (Toyooka et al. 2003). The lung microbiome exhibits distinct patterns based on cancer type and stage, with significant enrichment of *Proteobacteria* observed in lung cancer patients. Notably, microbial diversity is higher in patients with squamous cell carcinoma compared to those with adenocarcinoma, a trend particularly pronounced in men and heavy smokers (Morris et al. 2013). Studies in mice have demonstrated significant variations in lung microbiota, with no stable “core microbiota” detected in the lungs of healthy mice (Dickson et al. 2018). Additionally, other studies have reported lower microbial alpha diversity in tumor tissue compared to normal lung tissue, with specific bacteria such as *Acidovorax*, *Klebsiella*, *Rhodoferax*, and *Anaerococcus* (Goto. 2020) being more enriched in squamous cell carcinoma patients than in those with adenocarcinoma (Xu et al. 2020).

The dynamics of the lung microbiome play a pivotal role in modulating lung inflammation (Iwai et al. 2014; Segal et al. 2016), disease progression (Molyneaux et al. 2014; Han et al. 2014; Rogers et al. 2014), and the efficacy of various drug treatments (Goleva et al. 2013; Huang et al. 2011). Studies have revealed a bidirectional interaction between lung microbiota and the host (Dickson et al. 2016, 2017). Notably, an inverse correlation has been identified between lung microbiome diversity, quantified by the Shannon Diversity Index, and the concentrations of key inflammatory cytokines IL‐1α and IL‐4 (Dickson et al. 2018). Interestingly, this association is specific to the lung microbiome, as no such relationship has been found with the oral or cecal microbiota, suggesting that fluctuations in pulmonary cytokines are more directly linked to changes in the lung microbiota (Dickson et al. 2018). This finding underscores the potential of targeting the lung microbiome for the treatment of inflammatory lung conditions. Additionally, microbial imbalance within the lungs may contribute to lung cancer pathogenesis by disrupting metabolic pathways, modulating inflammation, and altering immune responses (Xu et al. 2020). Certain bacteria, prevalent in specific tumor types, possess metabolic pathways that degrade carcinogenic substances, such as those in cigarette smoke. For instance, in lung cancer, the MetaCyc pathway, which processes chemicals like toluene, acrylonitrile, and aminobenzoates, is significantly enriched in lung tumor‐associated bacteria. In smokers, lung tumors exhibit increased levels of *Proteobacteria*, *Actinobacteria*, and *Cyanobacteria*, suggesting that these bacteria thrive in environments rich in cigarette smoke metabolites, creating a conducive ecological niche. These intra‐tumoral microbes likely play a significant role in modulating tumor cell behavior and immune interactions (Sepich‐Poore et al. 2021; Nejman et al. 2020).

Previous studies on lung cancer microbiota have largely focused on individual‐level analyses, highlighting alpha and beta diversity in terms of species richness and community composition. However, the diversity scaling at the community and population levels across individuals has often been overlooked. Exploring lung tissue microbiome offers significant potential for advancing the prevention and treatment of lung cancer. Comprehensive investigations into the lung microbiome are essential for understanding its role in cancer progression and the variability in response to therapeutic interventions. Differences in microbiome diversity scaling within lung cancer tissues can uncover population‐level microbiome characteristics linked to cancer, thus deepening our understanding of the cancer‐microbiome relationship. Ma (2018a, 2018b, 2019a, 2019b) introduced the Diversity–Area Relationship (DAR) model, which extends the traditional Species‐Area Relationship (SAR) model by using the Hill number to measure diversity instead of species richness. The DAR model has been applied to map the biogeography of microbiomes across major human habitats (Ma 2018a, 2018b) and various disease‐associated microbial communities, including scaling analyses of global microbiome diversity in hot springs (Li and Ma 2019), gender‐based microbiome diversity differences across multiple sites (Ma and Li 2019), diversity scaling of the Chinese gut microbiome in relation to lifestyle (Xiao et al. 2021), and the microbiome of breast milk in healthy individuals (Chen et al. 2022).

In this study, we analyzed parameters such as pairwise diversity overlap (PDO), maximum accrued diversity (MAD), and the ratio of local diversity to global accrual diversity (LGD) to assess human lung cancer microbiome (LCM) diversity using DAR. This approach allowed for the examination of population‐level diversity from multiple angles, assessing its impact on lung cancer and mapping the biogeographic patterns of lung cancer‐associated microbes. Targeting features and pathways linked to the cancer‐related microbiome could offer insights into modulating tumor immunity and improving responses to immunotherapy.

## 2 Material and Methods

### 2.1 Bioinformatics Analysis of LCM

LCM datasets were obtained from five published studies, comprising a total of 575 human samples that met the following stringent inclusion criteria: (1) publicly available raw 16S rRNA gene‐sequencing data from lung tissues (both tumor and adjacent normal); (2) well‐annotated metadata distinguishing tissue types; (3) minimum sample size ≥ 50; (4) Illumina‐based sequencing with comparable read lengths; and (5) publication in high‐impact journals to ensure data quality. Five publicly available LCM datasets from diverse populations were analyzed: PRJNA303190 (Yu et al. 2016; European, Italia), PRJNA327258 (European, Italia), PRJNA320383 (K. L. Greathouse et al. 2018), PRJNA647170 (O. Kovaleva et al. 2020; European, Russia), and PRJNA680529 (N. Dumont‐Leblond et al. 2021; North American, Canadian), comprising 575 samples in total. Adjacent non‐tumorous lung tissues (NT; *n* = 372) served as the control group and included 214 samples from PRJNA303190, 44 from PRJNA327258, 61 from PRJNA320383, 24 from PRJNA647170, and 29 from PRJNA680529. Primary tumor tissues (PT; *n* = 203) comprised 56 samples from PRJNA303190, 50 from PRJNA327258, 42 from PRJNA320383, 26 from PRJNA647170, and 29 from PRJNA680529; among these, 60 were lung adenocarcinoma (LUAD: 6, 22, 0, 14, 18 per data set), 101 were lung squamous cell carcinoma (LUSC: 50, 28, 0, 12, 11 per data set), and the 42 PT samples from PRJNA320383 lacked histological subtype annotation. For each data set, operational taxonomic units (OTUs) were quantified and both total and mean sequencing reads per sample were reported (see Table 1). Raw 16S rRNA sequencing reads were downloaded from the NCBI database (https://www.ncbi.nlm.nih.gov/). Adapter removal and quality trimming were carried out with Trimmomatic v0.39. Taxonomic profiling was then performed using Kraken2 v2.1.2, and species‐level abundance estimates were obtained via Bracken v2.6 (Bayesian re‐estimation of Kraken assignments) against the 16S_Greengenes_k2db database (accessed March 25, 2020). Finally, KrakenTools and R v3.6.3 were used to convert and aggregate the results into usable OTU tables. A summary of the OTU tables for each of the five LCM datasets can be found in Table 1. For subsequent analyses, samples were categorized into four groups: NT for normal tissue from lung cancer patients, LUAD for LUAD, LUSC for LUSC, and PT for tumor tissue samples including LUAD, LUSC, and other types of lung cancer.

**Table 1:** A brief description of five LCM datasets and the OTU tables information for each group of lung cancer microbiome datasets.

Group	ID	Treatment	Sample size	Total sample size	Num. of species	Sum of reads	Mean of reads	Reference	Collection date	Country
Data set #1	PRJNA303190	LUAD	6	270	60	15,250	2542	G.Yu et al. (2016) *Genome Biology*	2016	Italia
		LUSC	50		160	213,428	4269			
		NT	214		446	1,211,358	5661			
		PT	56		177	228,678	4084			
Data set #2	PRJNA327258	LUAD	22	94	193	182,712	8305	No Information	2016	Italia
		LUSC	28		207	205,296	7332			
		NT	44		214	208,132	4730			
		PT	50		267	388,008	7760			
Data set #3	PRJNA320383	NT	61	103	185	59,807	980	K. L. Greathouse, et al. (2018) *Genome Biology*	1987–2010	—
		PT	42		74	17,588	419			
Data set #4	PRJNA647170	LUAD	14	50	203	592,857	42,347	O. Kovaleva et al. (2020) *Biomedicine*s	2015–2017	Russia
		LUSC	12		191	430,542	35,879			
		NT	24		295	1,125,521	46,897			
		PT	26		281	1,023,399	39,361			
Data set #5	PRJNA680529	LUAD	18	58	184	259,219	14,401	N. Dumont‐Leblond et al. (2021) *PLoS One*.	2020	Canada
		LUSC	11		165	184,729	16,794			
		NT	29		228	379,507	13,086			
		PT	29		232	443,948	15,309			
Pooled		LUAD	60	575	369	1,050,038	17,501	—	—	—
		LUSC	101		394	1,033,995	10,237			
		NT	372		610	2,984,325	8,022			
		PT	203		505	2,101,621	10,353			

Abbreviations: LUAD = lung adenocarcinoma, LUSC = lung squamous cell carcinoma, NT = lung normal tissue, PT = primary tumor tissue.

### 2.2 DAR Analysis

We calculated alpha diversity in the LCM data set using the Hill number (Hill 1973), a mathematical framework in ecology that measures community diversity by integrating both species richness (the number of distinct species) and the relative abundance of each species within the community. (Jost 2007; Chao et al. 2012; Chao, Chiu, et al. 2014; Chao, Gotelli, et al. 2014).

(1) Dq=∑i=1Spiq1/(1−q).

In this formula, the number of species is represented by *S*, the relative abundance of species *i* is denoted by *p*_*i*_, and the diversity order is expressed as *q*. The Hill number is undefined when *q* = 1, but as *q* approaches 1 indefinitely, there exists a limit of the following form:

(2) D1=limq→1Dq=exp−∑i=1Spilog(pi)

^0^ *D* corresponds to species richness; ^1^ *D* reflects the Shannon entropy, and ^2^ *D* denotes the inverse Simpson index, with the parameter *q* influencing the weighting of species abundance frequencies.

The DAR model expands upon the classical SAR model (Storch et al. 2012; Tjørve 2009; Tjørve and Tjørve 2008). Two models, DAR‐PL (power law) and DAR‐PLEC (exponential cutoff power law), were used to fit the datasets (Ma 2018a, 2018b, 2019a, 2019b). The power law DAR (PL‐DAR) model is defined as follows:

(3) Dq=cAz.

In this model, *qD* represents alpha diversity as quantified by Hill numbers, *A* denotes the area, while *c* and *z* serve as the heterogeneity parameters of the PL‐DAR model. The power law with an exponential cut‐off (PLEC‐DAR) model is defined as follows:

(4) D=cAzexp(dA)q,

where *d* represents the taper‐off parameter, typically a negative value, while exp(*dA*) denotes the exponential decay term. To estimate the parameters of the DAR model, the power law equation is linearly transformed.

(5) ln(D)=ln(c)+zln(A),

(6) ln(D)=ln(c)+zln(A)+dA.

The parameter *z* represents the slope of the log‐linear transformed PL model, analogous to its role in the traditional SAR, where it reflects the ratio of diversity accumulation to area expansion. Parameter *c* in the PL model corresponds to the initial species diversity in the first unit of area. Since the sequence of area units can affect parameter *c*, we addressed this technical issue by performing 100 random permutations of all samples from each cohort, fitting a DAR model to each permutation. The final parameter values were derived by averaging the results from these 100 fits.

### 2.3 The DAR Profile

Four DAR‐based profiles proposed by Ma (2018a, 2018b): (1) DAR profile, (2) PDO profile as overlap proportions, (3) maximal accrued diversity (MAD) profile as potential diversities, and (4) local‐to‐global diversity (LGD) profile. These four profiles can be used to outline the biogeographic map of the microbiome within lung tissues. The DAR profile is characterized by a sequence of *z*‐values (scaling parameters) derived from the PL‐DAR model (Equations 3 and 5), which represent various diversity levels (*q*) or the *z*–*q* relationship.

PDO (*g*) between two adjacent regions of equal size measures the proportion of new diversity in the second region, defined as

(7) $$g=2(D_{A}-D_{2A})/D_{A}=2-2^{z}.$$

The parameter *g* typically ranges from 0 to 1, with *z* acting as the scaling parameter in the PL‐DAR model. When *z* = 1, *g* = 0, indicating no overlap between the two regions, whereas *z* = 0 results in *g* = 1, signifying complete overlap. The PDO profile is represented as a series of *g*‐values corresponding to different diversity orders (*q*).

Based on the PLEC model (Equations (4) and (6)), the MAD of a group or population is derived as

(8) Max(Dq)=Dmaxq=c(−zd)zexp(−z)=cAmaxzexp(−z),

where *A*_max_ represents the total number of areas needed to achieve maximum diversity, and is given by

(9) $$A_{\max}=-z/d.$$

*D*_max_ quantifies the potential diversity, representing the total number of species within a population or cohort microbiome. MAD profile is characterized by a series of *D*_max_ values across various diversity orders (*q*).

The LGD profile (the ratio of local diversity to global accrual diversity) denotes the ratio of local diversity to global accrued diversity, allowing for the estimation of the contribution of a microbial community within a region in relation to the global scale.

(10) $$\mathrm{LGD}=c/D_{\max}.$$

Here, *c* represents a parameter of the PL‐DAR model (Equations (3) and (5)). The LGD profile is defined as by a sequence of LGD values across varying diversity orders (*q*).

### 2.4 Design Schemes for DAR Analysis

Initially, we fitted DAR models to each LCM data set and obtained 18 PL‐DAR models and 18 PLEC‐DAR models. Then we combined the five data sets into four groups NT, PT, LUAD, and LUSC according to the treatments, and obtained four PL‐DAR models and four PLEC‐DAR models. Finally, we combined all 575 samples to fit one PL‐DAR model and one PLEC‐DAR model. Additionally, a randomization test with 100 resampling iterations was performed to assess whether significant differences existed in the DAR model parameters across different groups.

## 3 Results

Through the DAR analysis, DAR‐PL and DAR‐PLEC models were fitted to each cohort in the seven groups of LCM datasets, culminating in all parameters including DAR, PDO, MAD, and LGD curves (see Supporting Information S1: Table S1). The results shown in Table 2 and Supporting Information S1: Table S1 include the diversity order of Hill numbers (*q*), DAR parameters [*z*, *c*, *d*, *g*, *A*_max_, *D*_max_, and LGD%], goodness‐of‐fit (*R* and *p*), and number of successes (*N*) for 100 models fit based on resampled data. Details of the seven LCM datasets are summarized in Table 2. DAR parameters calculated from the merged data, based on sample types, are also displayed in Table 2. Combining all 575 samples into a single analysis of the overall lung tissue microbiome, the DAR models were well‐fitted across the datasets (see Table 2). At *q* = 0, *p* values were < 0.001 and *R* > 0.95 in 100 model fits, confirming the robustness of both the DAR‐PL and DAR‐PLEC models. Similarly, at *q* = 1, *p* values were < 0.001 and *R* > 0.9, with successful fitting across 100 resampling. For *q* = 2, the success rate for both models were 100% (24/24), with average *R* values of 0.825 (*p* < 0.05) for the DAR‐PL model and 0.883 (*p* < 0.05) for the DAR‐PLEC model. At *q* = 3, the average *R* values were 0.778 for DAR‐PL and 0.848 for DAR‐PLEC, indicating that both models provided a good fit (*p* < 0.05).

**Table 2:** Fitting the DAR (diversity–area relationship) models of the human lung cancer microbiomes (LCM) all combined datasets (with 100 times of random permutations for microbiome samples) for human lung cancer microbiomes.

		Power law (PL)						PL with exponential cutoff (PLEC)								
Group	Diversity order	*z*	*c*	*R*	*g*	*p* value	*N*	*z*	*d*	*c*	*R*	*p* value	*N*	*A*_max_	*D*_max_	LGD (%)
NT	*q* = 0	0.463	37.864	0.977	0.621	0.000	100	0.602	−0.001	28.140	0.990	0.000	99	511	606	7.26
	*q* = 1	0.324	14.725	0.925	0.747	0.000	100	0.497	−0.002	11.061	0.964	0.000	92	425	116	17.32
	*q* = 2	0.255	8.881	0.818	0.804	0.000	99	0.450	−0.002	6.264	0.905	0.000	87	510	49	27.15
	*q* = 3	0.219	6.836	0.738	0.833	0.000	100	0.412	−0.002	5.060	0.856	0.000	84	619	32	33.80
PT	*q* = 0	0.533	30.386	0.968	0.552	0.000	100	0.709	−0.003	21.234	0.984	0.000	100	392	524	6.65
	*q* = 1	0.409	12.102	0.934	0.669	0.000	100	0.599	−0.003	8.799	0.961	0.000	91	243	114	13.57
	*q* = 2	0.356	7.072	0.880	0.716	0.000	100	0.538	−0.003	5.563	0.927	0.000	86	359	56	18.47
	*q* = 3	0.318	5.581	0.834	0.749	0.000	100	0.492	−0.003	4.754	0.889	0.000	81	1200	39	24.27
LUAD	*q* = 0	0.574	42.786	0.986	0.509	0.000	100	0.731	−0.008	29.864	0.993	0.000	91	146	427	9.43
	*q* = 1	0.479	18.662	0.953	0.600	0.000	100	0.706	−0.011	10.244	0.971	0.000	68	344	112	14.61
	*q* = 2	0.415	11.126	0.869	0.659	0.000	99	0.626	−0.012	6.743	0.906	0.000	65	68	46	25.71
	*q* = 3	0.374	8.776	0.824	0.697	0.000	94	0.539	−0.010	5.834	0.851	0.001	72	135	32	33.88
LUSC	*q* = 0	0.572	32.778	0.974	0.510	0.000	100	0.756	−0.006	21.360	0.985	0.000	91	209	426	7.77
	*q* = 1	0.450	14.217	0.934	0.629	0.000	100	0.637	−0.007	8.740	0.959	0.000	87	257	94	15.38
	*q* = 2	0.410	8.758	0.888	0.665	0.000	100	0.587	−0.007	5.433	0.921	0.000	79	146	47	21.28
	*q* = 3	0.388	7.113	0.865	0.686	0.000	99	0.556	−0.007	4.436	0.898	0.000	77	131	32	23.83
Human lung cancer microbiomes	*q* = 0	0.421	51.250	0.971	0.661	0.000	100	0.555	−0.001	3.476	0.987	0.000	100	689	670	32.323
	*q* = 1	0.282	22.807	0.914	0.783	0.000	100	0.434	−0.001	2.606	0.957	0.000	98	490	124	13.542
	*q* = 2	0.209	14.481	0.799	0.842	0.000	100	0.375	−0.001	2.108	0.890	0.000	96	487	52	8.235
	*q* = 3	0.176	11.496	0.716	0.869	0.000	100	0.336	−0.001	1.902	0.837	0.000	94	1934	36	6.698

**Figure 1:**
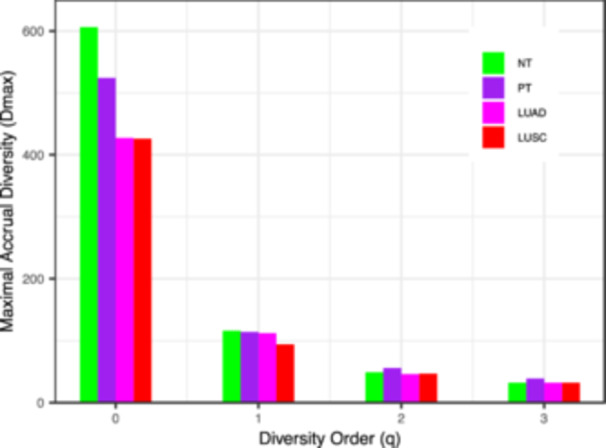
Maximal accrued diversity (MAD) profiles across diversity orders. Bar plots of estimated potential diversity *Dmax*(*q*) for four sample types—adjacent normal tissue (NT, green), all primary tumors (PT, purple), lung adenocarcinoma (LUAD, magenta), and lung squamous cell carcinoma (LUSC, red)—at diversity orders *q* = 0 (species richness), *q* = 1 (typical or common species), *q* = 2 (highly abundant species), and *q* = 3 (core or dominants species). Heights of the bars represent mean *D*_max_ values derived from 100 DAR‐PLEC model fits.

We performed pairwise comparisons of DAR parameters between different microbiome sample types in the same LCM data set (the results, see Supporting Information S1: Table S2). The results of the permutation tests showed that the parameters of the DAR analysis were very similar in comparison of the 13 normal and tumor tissues in the 5 LCM datasets. The results showed that in intra‐group comparisons, all the parameters of DAR analysis were not significant difference between the microbiome of normal tissues and different types of tumor tissues. Therefore, when we combined all samples, the *D*_max_ of NT is lower than PT at *q* = 0‐1. At *q* = 2–3, the *D*_max_ of PT is higher than NT (see Figure 1). We combined all the data used to calculate the DAR analysis parameters for the overall lung tissue microbial community of lung cancer patients. Our calculations indicated that the number of species that may be present in the microbiomes of lung tissues from lung cancer patients (potential species) could be up to 670, but there were only 124 typical species, 52 highly abundant species, and only 36 dominants species (*p *< 0.001). There are very few core species with stable microflora in the lung tissues of lung cancer patients.

**Figure 2:**
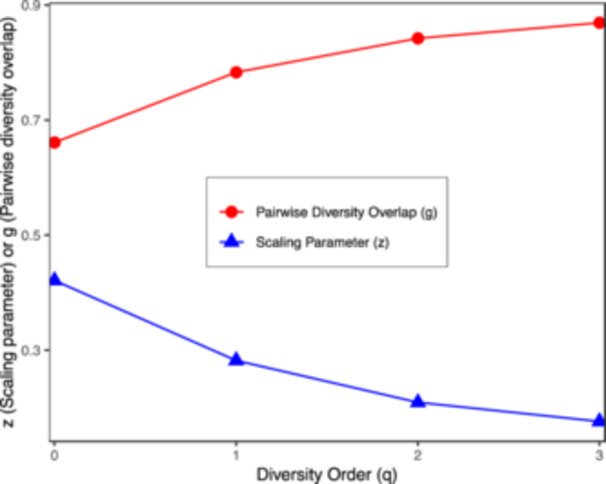
DAR and pairwise diversity overlap (PDO) profiles. Overlaid line plots (combined across all 575 samples) show how the DAR scaling exponent *z*(*q*) (blue triangles, blue solid line) and the pairwise overlap parameter *g*(*q*) (red circles, red solid line) vary with diversity order *q*. As *q* increases, *z* decreases while *g* increases, indicating slower accumulation of core species, reflecting greater community similarity among the most abundant species.

The scaling parameter *z* shows a consistent decrease as the diversity order *q* rises (Figure 2). Higher values of *z* represent faster changes in microbiome diversity among individuals in a population. The fastest change in species richness was observed in the LCM at *q* = 0, and the *z* value increased with decreasing *q* order. This suggests that while the total species count varied significantly between individuals, the diversity of core species remained relatively stable. In addition, the *z* values of NT were lower than those of PT, LUAD, or LUSC, which may indicate that the species diversity of NT is less variable. This demonstrates that the microbial community of normal tissues is more stable than that of tumor tissues. The overlap parameter *g*, which quantifies microbiome similarity, showed an inverse relationship to the DAR curve in Figure 2, with *g* values rising as the diversity order increased.

**Figure 3:**
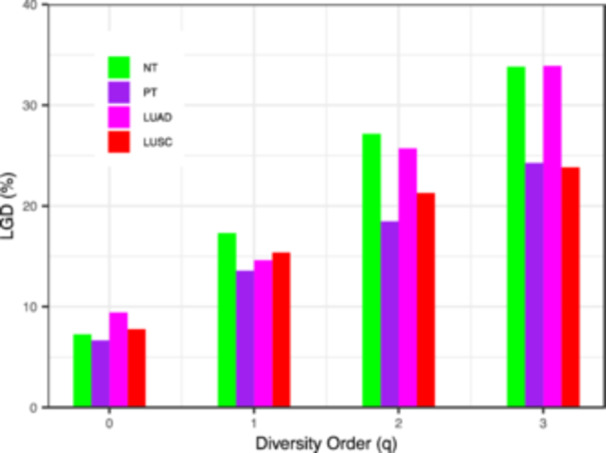
The ratio of local diversity to global accrual diversity profile (LGD‐*q*). The LGD profile plots, for each diversity order *q*, the ratio of mean local diversity (i.e., diversity in a single sample) to the maximal accrued diversity across all samples. In the bar chart, colors denote sample types: green for adjacent normal tissue (NT), purple for all primary tumors (PT), magenta for lung adenocarcinoma (LUAD), and red for lung squamous cell carcinoma (LUSC). The upward trend across *q* illustrates that increasingly abundant species account for a larger fraction of the total lung tissue microbiome.

LGD reflects the proportion of the microbial community in lung cancer patients’ tissue across different diversity orders *q*. The MAD and LGD profiles exhibit opposite patterns, with LGD increasing as *q* rises (see Figure 3). Individual microorganisms account for only 7.8% of the species in lung cancer tissue, while typical species contribute 19.3% and high‐abundance species represent 30.6% (*p *< 0.001). This indicates that species with greater abundance play a more critical role in maintaining the stability of the LCM. Parameter testing revealed no significant differences in LGD values between NT, PT, LUAD, or LUSC, suggesting that the proportions of common, rare, and dominant species were consistent across these communities.

In conclusion, by applying DAR‐PL and DAR‐PLEC models to 575 lung‐tissue microbiome samples (*q* = 0–3), we achieved highly robust model fits (*p* < 0.001, *R* > 0.95 at *q* = 0; *p* < 0.001, *R* > 0.900 at *q* = 1; 100% convergence with *p* < 0.001, average *R* > 0.800, at *q* = 2; *p* < 0.001, average *R* > 0.700 at *q* = 3). Pairwise comparisons of DAR parameters (*z*, *c*, *g*, *A*_max_, *D*_max_, LGD) between adjacent NT, PT, LUAD, and LUSC revealed no statistically significant differences, indicating invariant diversity–area scaling across tissues and individuals. The *z*‐value decreased with increasing diversity order *q*, reflecting faster turnover of rare species versus core species, while the overlap parameter *g* increased with *q*. MAD (*D*_max_) was estimated at ~670 species, of which 124 are common, 52 highly abundant, and 36 core species—individual species contributed 31%, 19%, and 9% of the total diversity, respectively. These results demonstrate a remarkable resilience of the lung microbiome to cancer‐associated disturbances.

## 4 Discussion

We analyzed the diversity scaling of 575 human LCM samples using the PL‐DAR and PLEC‐DAR models proposed by Ma (2018a, 2018b). Our study highlights the spatial heterogeneity and variation in community diversity across lung cancer tissue microbiomes, focusing on population‐level properties such as PDO, MAD, and the ratio of LGD. In the DAR profile, the *z*‐value reflects the ratio of diversity accumulation to area increase, indicating the rate of interindividual microbiome diversity change. A higher *z*‐value corresponds to faster shifts in diversity. The PDO profile's overlap parameter *g* measures the similarity between microbiomes in two regions of equal size. LGD estimates the proportion of microbial communities within a specific region compared to a global scale and provides insights into the diversity levels of lung cancer patients. This three‐parameter framework captures variations in community diversity and spatial heterogeneity. When the cumulative number of regions reaches *A*_max_, the MAD profile reflects the maximum diversity (*D*_max_) of all species within the population or cohort microbiome. Based on our calculations, lung tissue microbiomes of lung cancer patients may harbor up to 670 potential species, though only 124 are common, 52 are highly abundant, and 36 are core species. While individual microorganisms represent just 8% of the species, typical species contribute 19%, high‐abundance species 31%, and core species 37% (*p *< 0.001). Overall, diversity scaling parameters did not significantly differ between tissue types. It is important to note that these comparisons are based on species numbers rather than composition. Future investigations at the species and functional levels will be essential to identify specific microbial drivers of carcinogenesis and therapeutic response.

Our results reveal diversity scaling of the lung cancer‐associated microbiome at the population and community levels, which will require future insightful studies of 124 common, 52 highly abundant, and 36 core species, and determining the impact of interactions between different species on lung cancer progression is critical. Notably, the lack of significant differences in diversity scaling between normal and tumor tissues suggests that the microbiota's diversity variation is conserved across tissue types. Beyond diversity, microbiomes from various sources (such as gut, other mucosal, and intra‐tumoral microbes) can impact tumor behavior by influencing tumor growth, inflammatory response, immune evasion, genomic instability, and resistance to therapy (Knippel et al. 2021; Hanahan 2022; Ting et al. 2022). Microbes need mediators to foster tumor development, and it is widely believed that microbes affect host antitumor immunity through their derived metabolites, toxins, and antigens, rather than directly causing cancer. This relationship is referred to as microbial “Collusion.” The mechanisms of this “Collusion” are varied, including promoting tumor formation through inflammation, modifying immune and stromal cells in the tumor microenvironment, and facilitating immune escape via intestinal microbes (Hanahan and Weinberg 2011; Hanahan 2022). The influence of microbes on tumor behavior is thought to be indirect, with their derived metabolites, toxins, and antigens regulating tumor cell metabolism and remodeling the tumor microenvironment, thereby affecting tumor development and progression. This interaction between microbes and the tumor microenvironment is facilitated through the Immunology–oncology–microbiome (IOM) axis.

Despite the robustness of our diversity–area modeling, several limitations warrant consideration. First, the DAR framework provides only community‐level scaling metrics (e.g., exponents, overlap proportions, MAD and does not resolve the identities or functional roles of individual species; integrating differential‐abundance and core‐microbiome analyses will be necessary to pinpoint specific microbial drivers of lung tumor biology. Second, the five source cohorts exhibited methodological heterogeneity—including differences in DNA‐extraction protocols, primer sets (V3–V4 vs. V4 only), sequencing platforms (MiSeq vs. HiSeq), and read depths—which may influence low‐abundance species even after standardized reprocessing. Third, the reliance on 16S rRNA amplicon data precludes strain‐level resolution and functional characterization; shotgun metagenomics or meta‐transcriptomics would provide deeper insights into metabolic pathways and host–microbe interactions. Finally, limited clinical and demographic metadata (e.g., patient ethnicity, smoking history, tumor stage) across the public datasets constrained our ability to adjust for potential confounders. Addressing these gaps will strengthen future efforts to translate biogeographic diversity patterns into mechanistic understanding and therapeutic strategies.

Cancer‐associated microbes are increasingly used in diagnostics and treatment. Microbiota heterogeneity offers opportunities for disease diagnosis and localization, though challenges remain, including low microbial biomass and contamination risks. Questions about microbial stability and effectiveness during cancer treatment, particularly under antibiotic therapy, persist. Over 30 species have been detected in tumors, positioning microbes as potential diagnostic markers (Sepich‐Poore et al. 2021; Rooks and Garrett 2016). Additionally, the microbiome plays a critical role in modulating tumor treatment, with certain gastrointestinal microbiota affecting systemic lymphoid tissue and adjuvant immunotherapy outcomes (Ting et al. 2022). Intra‐tumoral microbiota is common and immunocompetent in most patients. For example, gut microbiota modulation alters the composition of intra‐tumoral microbiota in pancreatic cancer, while diet, medications, probiotics, and antibiotics can influence both gut and tumor microbiomes, with reciprocal effects on cancer therapies. Chemotherapy can alter the microbiome to enhance treatment effectiveness, whereas microbial degradation of chemicals can reduce therapeutic efficacy. Thus, the tumor microbiome plays a dual role in cancer therapy (Knippel et al. 2021; Nejman et al. 2020; Chowdhury et al. 2019).

Overall, our results provide the first description of diversity scaling in LCM. This foundational work paves the way for future studies that will focus on both the microbiome as a whole and individual microbial species to unlock deeper insights into their roles in cancer biology and treatment. Understanding the molecular mechanisms by which these microbes influence tumor growth and treatment outcomes will be critical for developing targeted microbial‐based therapies. Investigations into the stability of microbial communities during cancer treatment and their interaction with host immune responses could further refine personalized medicine approaches, enhancing therapeutic efficacy.

## Author Contributions

**Jiandong Mei:** writing – review and editing, data curation, conceptualization. **Yuting Qiao:** formal analysis, writing – original draft. **Zhanshan Ma:** writing – review and editing, supervision, methodology. All authors read and approved the final manuscript.

## Ethics Statement

The authors have nothing to report.

## Conflicts of Interest

The authors declare no conflicts of interest.

## Supporting information


**Table S1:** Fitting the DAR (diversity‐area relationship) models of the lung cancer microbiomes (LCM) datasets (with 100 times of random permutations for microbiome samples) for all microbiome sample types of 5 lung cancer microbiome datasets. **Table S2:** The results (percentages with significant differences) from the permutation tests for the differences in the DAR parameters with pair‐wise comparisons between different microbiome sample types of same lung cancer microbiome dataset.

## Data Availability

All data re‐analyzed in this study are available in the public domain. The raw sequencing data were collected from NCBI database (https://www.ncbi.nlm.nih.gov/). See Table 1 for detailed information.
