# Supplementary material for: Assessing Diversity Scaling in Lung Cancer Microbiome Across Individuals and Tissue Types
Source: Microbiologyopen. 2025 Aug 17;14(4):e70036. doi: 10.1002/mbo3.70036 (PMC12358694; doi:10.1002/mbo3.70036)
Supplement: Supplementary file 1 — Table S1: Fitting the DAR (diversity‐area relationship) models of the lung cancer microbiomes (LCM) datasets (with 100 times of random permutations for microbiome samples) for all microbiome sample types of 5 lung cancer microbiome datasets. Table S2: The results (percentages with significant differences) from the permutation tests for the differences in the DAR parameters with pair‐wise comparisons between different microbiome sample types of same lung cancer microbiome dataset. [file MBO3-14-e70036-s001.docx]

Assessing Diversity Scaling in Lung Cancer Microbiome Across Individuals and Tissue Types

**Online Supplementary Table (S1-S2)**

**Table S1**. Fitting the DAR (diversity-area relationship) models of the lung cancer microbiomes (LCM) datasets (with 100 times of random permutations for microbiome samples) for all microbiome sample types of 5 lung cancer microbiome datasets

**Table S2**. The results (percentages with significant differences) from the permutation tests for the differences in the DAR parameters with pair-wise comparisons between different microbiome sample types of same lung cancer microbiome dataset

**Table S1**. Fitting the DAR (diversity-area relationship) models of the lung cancer microbiomes (LCM) datasets (with 100 times of random permutations for microbiome samples) for all microbiome sample types of 5 lung cancer microbiome datasets

| **Cancer Disease** | **Tissue Site** | **Diversity Order** | **Power Law (PL)** | | | | | | **PL with Exponential Cutoff (PLEC)** | | | | | | | | |
| --- | --- | --- | --- | --- | --- | --- | --- | --- | --- | --- | --- | --- | --- | --- | --- | --- | --- |
|  |  |  | ***z*** | ***c*** | ***R*** | ***g*** | ***P*-value** | ***N*** | ***z*** | ***d*** | ***c*** | ***R*** | ***P*-value** | ***N*** | ***Amax*** | ***Dmax*** | ***LGD***  **(%)** |
| Dataset#1 | LUAD | *q* = 0 | 0.763 | 15.211 | 0.997 | 0.301 | 0.000 | 100 | 0.904 | -0.047 | 15.737 | 0.998 | 0.000 | 45 | 484 | 527 | 14.60 |
|  |  | *q* = 1 | 0.561 | 9.043 | 0.947 | 0.518 | 0.005 | 99 | 0.754 | -0.068 | 9.516 | 0.977 | 0.011 | 66 | 8 | 22.78 | 50.81 |
|  |  | *q* = 2 | 0.630 | 4.549 | 0.893 | 0.446 | 0.019 | 45 | 0.887 | -0.117 | 5.726 | 0.961 | 0.023 | 38 | 4 | 12.46 | 55.99 |
|  |  | *q* = 3 | 0.633 | 3.077 | 0.887 | 0.446 | 0.020 | 34 | 0.683 | -0.041 | 3.792 | 0.963 | 0.021 | 26 | 39 | 19.41 | 62.36 |
|  | LUSC | *q* = 0 | 0.630 | 14.571 | 0.978 | 0.448 | 0.000 | 100 | 0.814 | -0.012 | 11.577 | 0.987 | 0.000 | 83 | 109 | 181 | 9.02 |
|  |  | *q* = 1 | 0.447 | 7.591 | 0.905 | 0.631 | 0.000 | 100 | 0.624 | -0.012 | 6.443 | 0.937 | 0.000 | 61 | 84 | 46.32 | 22.10 |
|  |  | *q* = 2 | 0.378 | 4.816 | 0.773 | 0.692 | 0.000 | 97 | 0.341 | 0.000 | 5.749 | 0.819 | 0.001 | 62 | 62 | 18.95 | 42.42 |
|  |  | *q* = 3 | 0.356 | 3.815 | 0.735 | 0.713 | 0.002 | 92 | 0.241 | 0.005 | 4.933 | 0.806 | 0.000 | 61 | 122 | 13.88 | 46.08 |
|  | NT | *q* = 0 | 0.508 | 31.500 | 0.983 | 0.577 | 0.000 | 100 | 0.637 | -0.002 | 22.669 | 0.992 | 0.000 | 98 | 413 | 475 | 6.90 |
|  |  | *q* = 1 | 0.284 | 17.271 | 0.909 | 0.781 | 0.000 | 100 | 0.429 | -0.003 | 12.219 | 0.954 | 0.000 | 85 | 261 | 76.22 | 25.05 |
|  |  | *q* = 2 | 0.174 | 10.848 | 0.661 | 0.870 | 0.000 | 95 | 0.288 | -0.003 | 8.980 | 0.780 | 0.000 | 81 | 331 | 28.52 | 51.76 |
|  |  | *q* = 3 | 0.126 | 8.820 | 0.518 | 0.906 | 0.001 | 94 | 0.228 | -0.002 | 7.493 | 0.698 | 0.000 | 81 | 236 | 18.12 | 64.97 |
|  | PT | *q* = 0 | 0.612 | 16.151 | 0.981 | 0.468 | 0.000 | 100 | 0.789 | -0.010 | 12.441 | 0.988 | 0.000 | 84 | 193 | 227 | 8.16 |
|  |  | *q* = 1 | 0.433 | 8.348 | 0.910 | 0.643 | 0.000 | 100 | 0.619 | -0.012 | 6.896 | 0.940 | 0.000 | 68 | 92 | 49.65 | 22.15 |
|  |  | *q* = 2 | 0.372 | 5.302 | 0.789 | 0.696 | 0.000 | 99 | 0.427 | -0.006 | 5.795 | 0.844 | 0.000 | 60 | 65 | 22.72 | 37.50 |
|  |  | *q* = 3 | 0.348 | 4.263 | 0.742 | 0.718 | 0.001 | 97 | 0.325 | -0.002 | 5.058 | 0.796 | 0.001 | 64 | 39 | 15.16 | 45.00 |
| Dataset#2 | LUAD | *q* = 0 | 0.650 | 27.221 | 0.986 | 0.425 | 0.000 | 100 | 0.849 | -0.024 | 23.243 | 0.991 | 0.000 | 76 | 83 | 262 | 12.08 |
|  |  | *q* = 1 | 0.477 | 9.865 | 0.888 | 0.596 | 0.001 | 100 | 0.703 | -0.031 | 8.802 | 0.929 | 0.000 | 77 | 42 | 45.20 | 29.27 |
|  |  | *q* = 2 | 0.376 | 4.545 | 0.730 | 0.691 | 0.005 | 79 | 0.526 | -0.028 | 4.816 | 0.816 | 0.003 | 67 | 19 | 13.81 | 49.88 |
|  |  | *q* = 3 | 0.302 | 3.504 | 0.706 | 0.754 | 0.005 | 67 | 0.442 | -0.028 | 3.892 | 0.788 | 0.004 | 64 | 378 | 9.48 | 60.88 |
|  | LUSC | *q* = 0 | 0.605 | 28.905 | 0.980 | 0.472 | 0.000 | 100 | 0.875 | -0.025 | 21.520 | 0.987 | 0.000 | 60 | 75 | 251 | 12.58 |
|  |  | *q* = 1 | 0.341 | 9.403 | 0.827 | 0.725 | 0.001 | 89 | 0.538 | -0.024 | 8.585 | 0.878 | 0.000 | 73 | 42 | 28.54 | 45.16 |
|  |  | *q* = 2 | 0.103 | 5.646 | 0.708 | 0.910 | 0.003 | 73 | 0.246 | -0.017 | 5.223 | 0.773 | 0.003 | 75 | 20 | 8.39 | 90.68 |
|  |  | *q* = 3 | 0.046 | 4.527 | 0.710 | 0.953 | 0.004 | 70 | 0.154 | -0.013 | 4.354 | 0.773 | 0.003 | 75 | 29 | 5.77 | 110.41 |
|  | NT | *q* = 0 | 0.623 | 21.306 | 0.985 | 0.457 | 0.000 | 100 | 0.782 | -0.011 | 17.584 | 0.990 | 0.000 | 76 | 168 | 289 | 8.64 |
|  |  | *q* = 1 | 0.403 | 9.309 | 0.917 | 0.671 | 0.000 | 100 | 0.561 | -0.012 | 8.125 | 0.945 | 0.000 | 61 | 64 | 43.43 | 28.45 |
|  |  | *q* = 2 | 0.295 | 5.425 | 0.730 | 0.765 | 0.001 | 93 | 0.362 | -0.009 | 5.749 | 0.798 | 0.001 | 67 | 71 | 15.76 | 53.03 |
|  |  | *q* = 3 | 0.254 | 4.023 | 0.679 | 0.798 | 0.001 | 78 | 0.309 | -0.009 | 4.568 | 0.742 | 0.001 | 69 | 58 | 10.70 | 63.01 |
|  | PT | *q* = 0 | 0.578 | 30.024 | 0.983 | 0.505 | 0.000 | 100 | 0.747 | -0.011 | 24.047 | 0.992 | 0.000 | 94 | 126 | 310 | 10.35 |
|  |  | *q* = 1 | 0.353 | 10.475 | 0.856 | 0.716 | 0.001 | 100 | 0.601 | -0.016 | 7.599 | 0.914 | 0.000 | 76 | 65 | 41.09 | 30.27 |
|  |  | *q* = 2 | 0.196 | 4.988 | 0.673 | 0.846 | 0.003 | 81 | 0.324 | -0.011 | 4.855 | 0.736 | 0.001 | 79 | 71 | 11.58 | 66.90 |
|  |  | *q* = 3 | 0.123 | 4.145 | 0.643 | 0.903 | 0.005 | 78 | 0.239 | -0.010 | 3.995 | 0.722 | 0.002 | 74 | 36 | 7.18 | 82.63 |
| Dataset#3 | NT | *q* = 0 | 0.734 | 9.777 | 0.969 | 0.329 | 0.000 | 100 | 0.969 | -0.012 | 6.959 | 0.977 | 0.000 | 78 | 234 | 268 | 5.51 |
|  |  | *q* = 1 | 0.505 | 5.918 | 0.937 | 0.576 | 0.000 | 100 | 0.740 | -0.013 | 4.302 | 0.962 | 0.000 | 70 | 124 | 49.70 | 14.48 |
|  |  | *q* = 2 | 0.376 | 4.204 | 0.801 | 0.695 | 0.001 | 99 | 0.503 | -0.009 | 4.027 | 0.830 | 0.001 | 65 | 143 | 20.70 | 33.26 |
|  |  | *q* = 3 | 0.334 | 3.452 | 0.742 | 0.733 | 0.001 | 96 | 0.376 | -0.005 | 3.811 | 0.775 | 0.000 | 63 | 148 | 13.09 | 43.08 |
|  | PT | *q* = 0 | 0.708 | 5.714 | 0.975 | 0.361 | 0.000 | 100 | 0.976 | -0.019 | 4.100 | 0.986 | 0.000 | 82 | 110 | 89 | 7.24 |
|  |  | *q* = 1 | 0.519 | 3.751 | 0.895 | 0.558 | 0.000 | 100 | 0.745 | -0.019 | 3.184 | 0.931 | 0.000 | 65 | 63 | 25.82 | 20.72 |
|  |  | *q* = 2 | 0.423 | 2.863 | 0.775 | 0.651 | 0.001 | 96 | 0.452 | -0.005 | 3.108 | 0.843 | 0.000 | 69 | 44 | 12.55 | 37.37 |
|  |  | *q* = 3 | 0.387 | 2.394 | 0.732 | 0.686 | 0.001 | 88 | 0.344 | -0.001 | 2.930 | 0.790 | 0.001 | 68 | 108 | 9.15 | 46.44 |
| Dataset#4 | LUAD | *q* = 0 | 0.681 | 35.838 | 0.976 | 0.383 | 0.000 | 100 | 1.096 | -0.063 | 26.180 | 0.985 | 0.000 | 68 | 25 | 235 | 14.38 |
|  |  | *q* = 1 | 0.627 | 9.526 | 0.947 | 0.435 | 0.000 | 100 | 0.945 | -0.051 | 7.776 | 0.978 | 0.000 | 67 | 38 | 57.04 | 25.24 |
|  |  | *q* = 2 | 0.496 | 6.203 | 0.898 | 0.577 | 0.002 | 97 | 0.736 | -0.046 | 5.783 | 0.943 | 0.001 | 78 | 24 | 22.41 | 35.97 |
|  |  | *q* = 3 | 0.429 | 5.003 | 0.878 | 0.646 | 0.001 | 88 | 0.713 | -0.059 | 4.811 | 0.918 | 0.002 | 75 | 18 | 14.67 | 42.40 |
|  | LUSC | *q* = 0 | 0.697 | 36.379 | 0.964 | 0.362 | 0.000 | 100 | 1.142 | -0.089 | 30.846 | 0.981 | 0.000 | 72 | 56 | 303 | 18.27 |
|  |  | *q* = 1 | 0.592 | 10.486 | 0.891 | 0.468 | 0.003 | 92 | 0.810 | -0.050 | 10.299 | 0.927 | 0.004 | 74 | 14 | 41.94 | 37.62 |
|  |  | *q* = 2 | 0.584 | 6.271 | 0.912 | 0.486 | 0.001 | 79 | 0.613 | -0.020 | 7.092 | 0.918 | 0.005 | 69 | 17 | 25.89 | 42.94 |
|  |  | *q* = 3 | 0.541 | 5.436 | 0.893 | 0.534 | 0.003 | 78 | 0.676 | -0.037 | 5.675 | 0.929 | 0.003 | 61 | 15 | 19.81 | 40.14 |
|  | NT | *q* = 0 | 0.585 | 48.960 | 0.973 | 0.492 | 0.000 | 100 | 0.914 | -0.035 | 35.023 | 0.983 | 0.000 | 72 | 44 | 321 | 14.49 |
|  |  | *q* = 1 | 0.440 | 14.939 | 0.923 | 0.631 | 0.000 | 100 | 0.787 | -0.038 | 10.837 | 0.961 | 0.000 | 69 | 30 | 55.48 | 29.76 |
|  |  | *q* = 2 | 0.295 | 8.758 | 0.773 | 0.765 | 0.001 | 92 | 0.498 | -0.026 | 7.745 | 0.865 | 0.001 | 84 | 144 | 22.23 | 51.28 |
|  |  | *q* = 3 | 0.260 | 6.098 | 0.731 | 0.795 | 0.003 | 68 | 0.413 | -0.026 | 6.221 | 0.809 | 0.003 | 82 | 19 | 13.38 | 62.41 |
|  | PT | *q* = 0 | 0.616 | 39.845 | 0.975 | 0.462 | 0.000 | 100 | 0.909 | -0.029 | 29.488 | 0.986 | 0.000 | 72 | 66 | 323 | 12.46 |
|  |  | *q* = 1 | 0.535 | 11.519 | 0.951 | 0.542 | 0.000 | 100 | 0.851 | -0.030 | 8.199 | 0.979 | 0.000 | 69 | 44 | 66.38 | 17.69 |
|  |  | *q* = 2 | 0.413 | 7.877 | 0.873 | 0.661 | 0.000 | 100 | 0.681 | -0.028 | 6.197 | 0.944 | 0.000 | 77 | 35 | 28.74 | 31.68 |
|  |  | *q* = 3 | 0.328 | 6.619 | 0.787 | 0.740 | 0.002 | 92 | 0.591 | -0.032 | 5.579 | 0.877 | 0.001 | 84 | 27 | 18.38 | 43.04 |
| Dataset#5 | LUAD | *q* = 0 | 0.616 | 33.082 | 0.979 | 0.463 | 0.000 | 100 | 0.864 | -0.036 | 28.219 | 0.989 | 0.000 | 82 | 102 | 302 | 16.03 |
|  |  | *q* = 1 | 0.579 | 18.954 | 0.965 | 0.500 | 0.000 | 100 | 0.903 | -0.049 | 15.658 | 0.985 | 0.000 | 87 | 27 | 98.05 | 20.27 |
|  |  | *q* = 2 | 0.538 | 14.454 | 0.946 | 0.541 | 0.000 | 100 | 0.901 | -0.055 | 11.811 | 0.977 | 0.000 | 87 | 22 | 64.47 | 23.96 |
|  |  | *q* = 3 | 0.511 | 12.110 | 0.926 | 0.567 | 0.000 | 100 | 0.863 | -0.055 | 10.085 | 0.965 | 0.000 | 87 | 20 | 49.12 | 28.19 |
|  | LUSC | *q* = 0 | 0.599 | 41.388 | 0.991 | 0.484 | 0.000 | 100 | 0.800 | -0.047 | 39.766 | 0.997 | 0.000 | 92 | 45 | 212 | 21.79 |
|  |  | *q* = 1 | 0.540 | 22.624 | 0.978 | 0.543 | 0.000 | 100 | 0.821 | -0.064 | 21.413 | 0.993 | 0.000 | 87 | 21 | 83.70 | 28.271 |
|  |  | *q* = 2 | 0.485 | 15.768 | 0.939 | 0.592 | 0.000 | 100 | 0.775 | -0.066 | 14.850 | 0.971 | 0.000 | 84 | 33 | 53.45 | 36.49 |
|  |  | *q* = 3 | 0.453 | 12.528 | 0.885 | 0.620 | 0.003 | 95 | 0.766 | -0.071 | 11.681 | 0.942 | 0.002 | 73 | 9 | 32.91 | 43.01 |
|  | NT | *q* = 0 | 0.625 | 30.205 | 0.978 | 0.452 | 0.000 | 100 | 0.845 | -0.022 | 24.386 | 0.988 | 0.000 | 87 | 77 | 277 | 12.21 |
|  |  | *q* = 1 | 0.497 | 18.247 | 0.959 | 0.585 | 0.000 | 100 | 0.740 | -0.026 | 14.820 | 0.981 | 0.000 | 94 | 40 | 90.96 | 21.27 |
|  |  | *q* = 2 | 0.392 | 14.041 | 0.907 | 0.685 | 0.000 | 100 | 0.677 | -0.030 | 10.935 | 0.957 | 0.000 | 92 | 37 | 49.88 | 29.56 |
|  |  | *q* = 3 | 0.327 | 11.965 | 0.844 | 0.742 | 0.001 | 100 | 0.645 | -0.033 | 9.070 | 0.925 | 0.000 | 88 | 54 | 35.67 | 35.88 |
|  | PT | *q* = 0 | 0.552 | 38.939 | 0.985 | 0.531 | 0.000 | 100 | 0.725 | -0.018 | 33.482 | 0.994 | 0.000 | 94 | 114 | 289 | 15.39 |
|  |  | *q* = 1 | 0.480 | 22.737 | 0.967 | 0.602 | 0.000 | 100 | 0.714 | -0.025 | 18.746 | 0.988 | 0.000 | 97 | 41 | 106.17 | 22.63 |
|  |  | *q* = 2 | 0.417 | 17.219 | 0.934 | 0.661 | 0.000 | 100 | 0.699 | -0.030 | 13.667 | 0.974 | 0.000 | 92 | 31 | 63.74 | 28.84 |
|  |  | *q* = 3 | 0.370 | 14.527 | 0.885 | 0.703 | 0.000 | 100 | 0.666 | -0.032 | 11.508 | 0.946 | 0.000 | 92 | 30 | 46.79 | 34.54 |
| Pooled | LUAD | *q* = 0 | 0.574 | 42.786 | 0.986 | 0.509 | 0.000 | 100 | 0.731 | -0.008 | 29.864 | 0.993 | 0.000 | 91 | 146 | 427 | 9.43 |
|  |  | *q* = 1 | 0.479 | 18.662 | 0.953 | 0.600 | 0.000 | 100 | 0.706 | -0.011 | 10.244 | 0.971 | 0.000 | 68 | 344 | 112 | 14.61 |
|  |  | *q* = 2 | 0.415 | 11.126 | 0.869 | 0.659 | 0.000 | 99 | 0.626 | -0.012 | 6.743 | 0.906 | 0.000 | 65 | 68 | 46 | 25.71 |
|  |  | *q* = 3 | 0.374 | 8.776 | 0.824 | 0.697 | 0.000 | 94 | 0.539 | -0.010 | 5.834 | 0.851 | 0.001 | 72 | 135 | 32 | 33.88 |
|  | LUSC | *q* = 0 | 0.572 | 32.778 | 0.974 | 0.510 | 0.000 | 100 | 0.756 | -0.006 | 21.360 | 0.985 | 0.000 | 91 | 209 | 426 | 7.77 |
|  |  | *q* = 1 | 0.450 | 14.217 | 0.934 | 0.629 | 0.000 | 100 | 0.637 | -0.007 | 8.740 | 0.959 | 0.000 | 87 | 257 | 94 | 15.38 |
|  |  | *q* = 2 | 0.410 | 8.758 | 0.888 | 0.665 | 0.000 | 100 | 0.587 | -0.007 | 5.433 | 0.921 | 0.000 | 79 | 146 | 47 | 21.28 |
|  |  | *q* = 3 | 0.388 | 7.113 | 0.865 | 0.686 | 0.000 | 99 | 0.556 | -0.007 | 4.436 | 0.898 | 0.000 | 77 | 131 | 32 | 23.83 |
|  | NT | *q* = 0 | 0.463 | 37.864 | 0.977 | 0.621 | 0.000 | 100 | 0.602 | -0.001 | 28.140 | 0.990 | 0.000 | 99 | 511 | 606 | 7.26 |
|  |  | *q* = 1 | 0.324 | 14.725 | 0.925 | 0.747 | 0.000 | 100 | 0.497 | -0.002 | 11.061 | 0.964 | 0.000 | 92 | 425 | 116 | 17.32 |
|  |  | *q* = 2 | 0.255 | 8.881 | 0.818 | 0.804 | 0.000 | 99 | 0.450 | -0.002 | 6.264 | 0.905 | 0.000 | 87 | 510 | 49 | 27.15 |
|  |  | *q* = 3 | 0.219 | 6.836 | 0.738 | 0.833 | 0.000 | 100 | 0.412 | -0.002 | 5.060 | 0.856 | 0.000 | 84 | 619 | 32 | 33.80 |
|  | PT | *q* = 0 | 0.533 | 30.386 | 0.968 | 0.552 | 0.000 | 100 | 0.709 | -0.003 | 21.234 | 0.984 | 0.000 | 100 | 392 | 524 | 6.65 |
|  |  | *q* = 1 | 0.409 | 12.102 | 0.934 | 0.669 | 0.000 | 100 | 0.599 | -0.003 | 8.799 | 0.961 | 0.000 | 91 | 243 | 114 | 13.57 |
|  |  | *q* = 2 | 0.356 | 7.072 | 0.880 | 0.716 | 0.000 | 100 | 0.538 | -0.003 | 5.563 | 0.927 | 0.000 | 86 | 359 | 56 | 18.47 |
|  |  | *q* = 3 | 0.318 | 5.581 | 0.834 | 0.749 | 0.000 | 100 | 0.492 | -0.003 | 4.754 | 0.889 | 0.000 | 81 | 1200 | 39 | 24.27 |

**Table S2**. The results (percentages with significant differences) from the permutation tests for the differences in the DAR parameters with pair-wise comparisons between different microbiome sample types of same lung cancer microbiome dataset

| **Dataset** | **Treatment** | **Diversity order** | **PL** | | **PLEC** | | | | | |
| --- | --- | --- | --- | --- | --- | --- | --- | --- | --- | --- |
|  |  |  | ***z*** | **ln(*c*)** | ***z*** | ***d*** | **ln(*c*)** | ***A_max_*** | ***D_max_*** | ***LGD*** |
| Dataset#1 | LUAD *vs*. LUSC | *q* = 0 | 0.652 | 0.981 | 0.850 | 0.844 | 0.636 | 0.218 | 0.810 | 0.755 |
|  |  | *q* = 1 | 0.782 | 0.780 | 0.872 | 0.820 | 0.592 | 0.158 | 0.702 | 0.590 |
|  |  | *q* = 2 | 0.586 | 0.933 | 0.559 | 0.692 | 0.821 | 0.435 | 0.769 | 0.912 |
|  |  | *q* = 3 | 0.503 | 0.880 | 0.613 | 0.819 | 0.699 | 0.392 | 0.757 | 0.890 |
|  | NT *vs*. LUAD | *q* = 0 | 0.487 | 0.380 | 0.688 | 0.811 | 0.556 | 0.097 | 0.497 | 0.742 |
|  |  | *q* = 1 | 0.544 | 0.540 | 0.636 | 0.756 | 0.769 | 0.118 | 0.397 | 0.498 |
|  |  | *q* = 2 | 0.364 | 0.500 | 0.495 | 0.704 | 0.636 | 0.196 | 0.486 | 0.946 |
|  |  | *q* = 3 | 0.293 | 0.398 | 0.584 | 0.848 | 0.546 | 0.195 | 0.540 | 0.970 |
|  | NT *vs*. LUSC | *q* = 0 | 0.554 | 0.244 | 0.549 | 0.485 | 0.321 | 0.212 | 0.173 | 0.798 |
|  |  | *q* = 1 | 0.466 | 0.274 | 0.553 | 0.581 | 0.404 | 0.223 | 0.212 | 0.898 |
|  |  | *q* = 2 | 0.465 | 0.390 | 0.947 | 0.789 | 0.785 | 0.288 | 0.410 | 0.922 |
|  |  | *q* = 3 | 0.484 | 0.440 | 0.865 | 0.608 | 0.806 | 0.338 | 0.593 | 0.819 |
|  | NT *vs*. PT | *q* = 0 | 0.494 | 0.219 | 0.487 | 0.424 | 0.293 | 0.561 | 0.470 | 0.857 |
|  |  | *q* = 1 | 0.431 | 0.301 | 0.566 | 0.517 | 0.437 | 0.187 | 0.190 | 0.958 |
|  |  | *q* = 2 | 0.423 | 0.414 | 0.842 | 0.998 | 0.661 | 0.435 | 0.671 | 0.784 |
|  |  | *q* = 3 | 0.404 | 0.453 | 0.988 | 0.763 | 0.767 | 0.577 | 0.931 | 0.769 |
| Dataset#2 | LUAD *vs*. LUSC | *q* = 0 | 0.933 | 0.964 | 0.956 | 0.881 | 0.925 | 0.416 | 0.493 | 0.899 |
|  |  | *q* = 1 | 0.624 | 0.898 | 0.796 | 0.905 | 0.836 | 0.674 | 0.155 | 0.749 |
|  |  | *q* = 2 | 0.498 | 0.954 | 0.642 | 0.842 | 0.962 | 0.741 | 0.376 | 0.629 |
|  |  | *q* = 3 | 0.545 | 0.946 | 0.642 | 0.786 | 0.939 | 0.335 | 0.384 | 0.704 |
|  | NT *vs*. LUAD | *q* = 0 | 0.847 | 0.683 | 0.824 | 0.673 | 0.707 | 0.669 | 0.644 | 0.711 |
|  |  | *q* = 1 | 0.829 | 0.881 | 0.808 | 0.707 | 0.831 | 0.544 | 0.844 | 0.767 |
|  |  | *q* = 2 | 0.825 | 0.871 | 0.874 | 0.804 | 0.992 | 0.244 | 0.825 | 0.879 |
|  |  | *q* = 3 | 0.931 | 0.923 | 0.921 | 0.798 | 0.988 | 0.240 | 0.885 | 0.864 |
|  | NT *vs*. LUSC | *q* = 0 | 0.925 | 0.721 | 0.858 | 0.716 | 0.802 | 0.785 | 0.893 | 0.778 |
|  |  | *q* = 1 | 0.748 | 0.971 | 0.967 | 0.742 | 0.956 | 0.409 | 0.311 | 0.566 |
|  |  | *q* = 2 | 0.611 | 0.900 | 0.742 | 0.938 | 0.971 | 0.259 | 0.189 | 0.494 |
|  |  | *q* = 3 | 0.552 | 0.973 | 0.714 | 0.972 | 0.954 | 0.436 | 0.230 | 0.530 |
|  | NT *vs*. PT | *q* = 0 | 0.666 | 0.418 | 0.782 | 0.849 | 0.486 | 0.722 | 0.404 | 0.767 |
|  |  | *q* = 1 | 0.713 | 0.784 | 0.954 | 0.881 | 0.875 | 0.561 | 0.977 | 0.779 |
|  |  | *q* = 2 | 0.580 | 0.939 | 0.775 | 0.992 | 0.953 | 0.526 | 0.373 | 0.589 |
|  |  | *q* = 3 | 0.550 | 0.948 | 0.783 | 0.975 | 0.994 | 0.921 | 0.405 | 0.532 |
| Dataset#3 | NT *vs*. PT | *q* = 0 | 0.604 | 0.710 | 0.804 | 0.844 | 0.788 | 0.458 | 0.129 | 0.505 |
|  |  | *q* = 1 | 0.839 | 0.741 | 0.912 | 0.817 | 0.892 | 0.371 | 0.146 | 0.353 |
|  |  | *q* = 2 | 0.928 | 0.756 | 0.865 | 0.982 | 0.991 | 0.263 | 0.400 | 0.647 |
|  |  | *q* = 3 | 0.908 | 0.801 | 0.875 | 0.966 | 0.957 | 0.053 | 0.608 | 0.697 |
| Dataset#4 | LUAD *vs*. LUSC | *q* = 0 | 0.977 | 0.970 | 0.962 | 0.833 | 0.897 | 0.600 | 0.926 | 0.758 |
|  |  | *q* = 1 | 0.935 | 0.922 | 0.716 | 0.787 | 0.733 | 0.330 | 0.578 | 0.464 |
|  |  | *q* = 2 | 0.894 | 0.919 | 0.645 | 0.578 | 0.694 | 0.427 | 0.641 | 0.660 |
|  |  | *q* = 3 | 0.752 | 0.927 | 0.684 | 0.595 | 0.661 | 0.890 | 0.585 | 0.846 |
|  | NT *vs*. LUAD | *q* = 0 | 0.629 | 0.568 | 0.666 | 0.559 | 0.662 | 0.693 | 0.464 | 0.939 |
|  |  | *q* = 1 | 0.531 | 0.547 | 0.743 | 0.764 | 0.661 | 0.217 | 0.749 | 0.861 |
|  |  | *q* = 2 | 0.500 | 0.627 | 0.651 | 0.731 | 0.672 | 0.177 | 0.911 | 0.697 |
|  |  | *q* = 3 | 0.521 | 0.706 | 0.668 | 0.718 | 0.726 | 0.897 | 0.910 | 0.709 |
|  | NT *vs*. LUSC | *q* = 0 | 0.618 | 0.586 | 0.612 | 0.436 | 0.773 | 0.956 | 0.545 | 0.778 |
|  |  | *q* = 1 | 0.628 | 0.600 | 0.931 | 0.928 | 0.931 | 0.099 | 0.390 | 0.594 |
|  |  | *q* = 2 | 0.425 | 0.705 | 0.913 | 0.715 | 0.969 | 0.268 | 0.587 | 0.947 |
|  |  | *q* = 3 | 0.360 | 0.778 | 0.947 | 0.727 | 0.940 | 0.775 | 0.395 | 0.800 |
|  | LUSC *vs*. PT | *q* = 0 | 0.742 | 0.855 | 0.621 | 0.416 | 0.955 | 0.735 | 0.327 | 0.563 |
|  |  | *q* = 1 | 0.837 | 0.871 | 0.890 | 0.990 | 0.866 | 0.590 | 0.417 | 0.460 |
|  |  | *q* = 2 | 0.675 | 0.806 | 0.841 | 0.770 | 0.927 | 0.881 | 0.968 | 0.775 |
|  |  | *q* = 3 | 0.518 | 0.761 | 0.908 | 0.797 | 0.929 | 0.802 | 0.816 | 0.956 |
| Dataset#5 | LUAD *vs*. LUSC | *q* = 0 | 0.891 | 0.567 | 0.824 | 0.883 | 0.401 | 0.764 | 0.919 | 0.536 |
|  |  | *q* = 1 | 0.847 | 0.703 | 0.864 | 0.813 | 0.520 | 0.099 | 0.205 | 0.384 |
|  |  | *q* = 2 | 0.828 | 0.839 | 0.919 | 0.786 | 0.648 | 0.719 | 0.492 | 0.424 |
|  |  | *q* = 3 | 0.849 | 0.950 | 0.932 | 0.814 | 0.763 | 0.655 | 0.383 | 0.494 |
|  | NT *vs*. LUAD | *q* = 0 | 0.881 | 0.988 | 0.864 | 0.712 | 0.977 | 0.519 | 0.549 | 0.813 |
|  |  | *q* = 1 | 0.595 | 0.922 | 0.637 | 0.619 | 0.913 | 0.242 | 0.494 | 0.702 |
|  |  | *q* = 2 | 0.403 | 0.912 | 0.525 | 0.608 | 0.903 | 0.535 | 0.249 | 0.485 |
|  |  | *q* = 3 | 0.353 | 0.883 | 0.511 | 0.651 | 0.898 | 0.927 | 0.209 | 0.437 |
|  | NT *vs*. LUSC | *q* = 0 | 0.982 | 0.682 | 0.974 | 0.735 | 0.509 | 0.643 | 0.856 | 0.426 |
|  |  | *q* = 1 | 0.778 | 0.798 | 0.806 | 0.602 | 0.642 | 0.232 | 0.605 | 0.672 |
|  |  | *q* = 2 | 0.708 | 0.952 | 0.733 | 0.574 | 0.769 | 0.471 | 0.817 | 0.900 |
|  |  | *q* = 3 | 0.654 | 0.940 | 0.732 | 0.616 | 0.879 | 0.806 | 0.952 | 0.991 |
|  | NT *vs*. PT | *q* = 0 | 0.749 | 0.694 | 0.762 | 0.851 | 0.682 | 0.567 | 0.801 | 0.737 |
|  |  | *q* = 1 | 0.988 | 0.741 | 0.992 | 0.989 | 0.793 | 0.251 | 0.981 | 0.991 |
|  |  | *q* = 2 | 0.819 | 0.779 | 0.887 | 0.979 | 0.825 | 0.549 | 0.378 | 0.755 |
|  |  | *q* = 3 | 0.767 | 0.824 | 0.865 | 0.971 | 0.835 | 0.313 | 0.437 | 0.770 |
| Total Percentage (%) with Significant Difference | | *q* = 0 | 0 | 0 | 0 | 0 | 0 | 0 | 0 | 0 |
|  |  | *q* = 1 | 0 | 0 | 0 | 0 | 0 | 0 | 0 | 0 |
|  |  | *q* = 2 | 0 | 0 | 0 | 0 | 0 | 0 | 0 | 0 |
|  |  | *q* = 3 | 0 | 0 | 0 | 0 | 0 | 0 | 0 | 0 |
